# *Codium fragile* Extract Ameliorates Respiratory Function by Controlling Allergic Inflammation in Ovalbumin-Induced Bronchial Disorders in Mice

**DOI:** 10.3390/md23050221

**Published:** 2025-05-21

**Authors:** Hyo Lim Lee, Yeong Hyeon Ju, In Young Kim, Hye Ji Choi, Yu Mi Heo, Hwa Rang Na, Ho Jin Heo

**Affiliations:** Division of Applied Life Science (BK21), Institute of Agriculture and Life Science, Gyeongsang National University, Jinju 52828, Republic of Korea; gyfla059@gnu.ac.kr (H.L.L.); ju8172001@gnu.ac.kr (Y.H.J.); inzero331@gnu.ac.kr (I.Y.K.); hjchoi0820@gnu.ac.kr (H.J.C.); yumi@gnu.ac.kr (Y.M.H.); hrna@gnu.ac.kr (H.R.N.)

**Keywords:** *Codium fragile*, oleamide, allergic inflammation, Th2 cytokine, fibrosis, pulmonary function

## Abstract

This study investigated the effect of *Codium fragile* (WCF) water extract in reducing allergic inflammation in ovalbumin (OVA)-induced mice. Mice were sensitized to OVA + aluminum hydroxide, administered WCF for one week, and exposed to 1% aerosolized OVA. As a result, WCF intake reduced the OVA-induced increase in CD4^+^ T cells, CD8^+^ T cells, the T helper type 2 (Th2)/T helper type 1 (Th1) cell ratio, and inflammatory cells such as eosinophils and lymphocytes. Furthermore, WCF reduced Th2 cytokines such as interleukin (IL)-5, IL-13, and IL-33 and inflammatory cytokines such as tumor necrosis factor α (TNF-α) and IL-1β in lung tissues. A histological analysis showed that WCF intake decreases OVA-induced pulmonary inflammation, bronchial wall thickness, and mucus score and increases pulmonary alveolar area. Moreover, WCF inhibited the nuclear factor κB (NF-κB) pathway, the transforming growth factor β (TGF-β)/Smad pathway, and apoptosis-related proteins in lung tissues that OVA excessively activated. The oleamide (9-octadecenamide) content, representing a physiologically active component of WCF, was analyzed and validated using a high-performance liquid chromatography-photodiode array (HPLC-PDA) system. These results demonstrate that WCF may serve as a potential preventive agent for respiratory dysfunction such as allergic asthma by suppressing NF-κB and TGF-β/Smad pathways.

## 1. Introduction

A recent chronic respiratory disease (CRD) morbidity and mortality study reported that non-communicable diseases, such as respiratory diseases, cardiovascular diseases, and neurological diseases, accounted for 73.4% of total deaths [1]. Respiratory diseases, including asthma and chronic obstructive pulmonary disease (COPD), affect several pulmonary components, including the airways and lungs, thus resulting in abnormal physiological function [2]. Globally, asthma is one of the most common chronic diseases in which airway inflammation, the hypersecretion of mucus, airway remodeling, and leukocyte infiltration are observed, which are related to a decline in lung function [3]. Used as representative clinical indicators, T helper type 2 (Th2) cytokines such as interleukin (IL)-4, IL-5, and IL-13 secreted by Th2 cells play an important role in the pathogenesis of eosinophilic asthma [4]. Th2 immune hyperactivation is a distinct characteristic of asthma and promotes eosinophil activation, increased immunoglobulin (Ig)E-mediated allergic responses, and fibrosis [4,5]. Various treatment approaches are available to alleviate complications associated with respiratory inflammation, including oxygen therapy, steam inhalation, mucus drainage, and the use of antihistamines [6]. Pharmacological agents commonly prescribed to manage asthma symptoms include β_2_-adrenoceptor agonists, inhaled corticosteroids, leukotriene receptor antagonists such as montelukast, muscarinic antagonists (M-cholinolytics), phosphodiesterase inhibitors, lipoxygenase (LOX) inhibitors, and mast cell stabilizers. However, long-term use of these agents has been associated with adverse effects [7]. In addition, biologics targeting IgE and interleukins, such as omalizumab, mepolizumab, benralizumab, and dupilumab, have emerged as effective therapies for allergic diseases, including asthma [8]. Despite the clinical efficacy of these biologics, their high cost and the risk of symptom recurrence after discontinuation highlight the need for alternative strategies to ensure sustained asthma control [9]. Therefore, it is necessary to research phytochemicals as anti-inflammatory agents to discover safe and effective treatments for asthma. The use of plant products in the treatment of asthma has been reported in traditional medicine for over 5000 years [10]. The most studied plant-derived compounds are alkaloids, stilbenes, flavonoids, glycosides, and saponins, which have been shown to have activity against anti-inflammatory properties with respiratory diseases [6].

The marine environment, with its vast biodiversity, is a rich source of natural compounds. For this reason, numerous studies have investigated the antimicrobial, antioxidant, anti-inflammatory, and anti-asthmatic properties of various algae and marine natural products [11]. *Codium fragile* (*C. fragile*) is a type of seaweed commonly consumed in Korea belonging to the *Codiaceae* family. It is found in many countries around the world, including Korea, Japan, and North America [12]. Several in vitro and in vivo studies reported that *C. fragile* has antioxidant and anti-inflammatory effects [13,14]. Moreover, it has been reported by previous studies that oleamide (9-octadecenamide), known as an indicator component and effective ingredient of *C. fragile*, has strong anti-allergic, anti-inflammatory, and antioxidant effects [15,16,17]. Utilizing this knowledge as a foundation, we preliminarily confirmed through a preliminary study that water extract of *C. fragile* improves particulate matter-induced pulmonary dysfunction [12]. However, the improvement effect of *C. fragile* on ovalbumin (OVA)-induced allergy inflammation and lung damage has not yet been elucidated. Therefore, this study was conducted to investigate the effects of the hot water extract of *C. fragile* (WCF) on respiratory function in mice with OVA-induced allergic bronchial disorders. Furthermore, a method was developed for analyzing the indicator component, oleamide, in WCF for industrial applications. The reliability of this analytical method was confirmed through validation.

## 2. Results

### 2.1. High-Performance Liquid Chromatography with Photodiode Array Detection (HPLC-PDA) Analysis

#### 2.1.1. Quantitative Analysis of Oleamide in WCF

**Figure 1:**
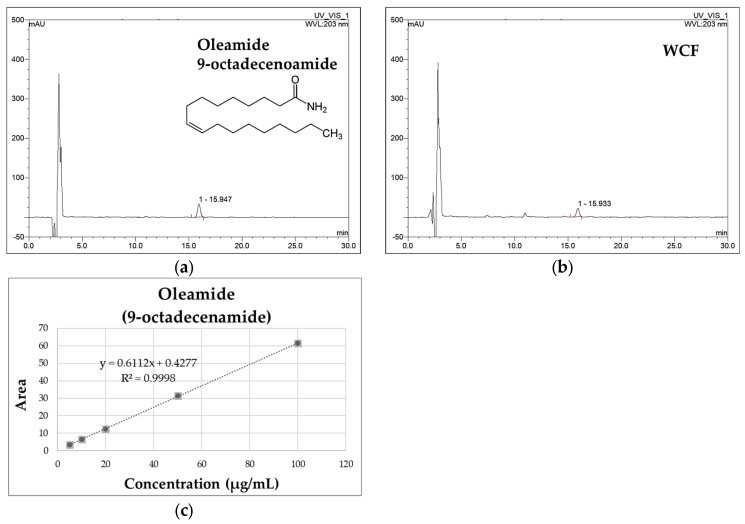
The HPLC chromatograms of oleamide standard (**a**) and water extract of *Codium fragile* (WCF) (**b**). The calibration curve of oleamide (**c**).

WCF and oleamide HPLC chromatograms are presented in Figure 1. The oleamide standard compound was identified at 15.930 min. The peak at 15.943 min in the chromatogram of WCF showed a similarity index of 0.999 with oleamide. A calibration curve was created using the linear relationships between the peak area and concentration of the standard solution. Based on this, the concentration of oleamide in WCF was calculated as 3.85 ± 0.06 μg/mg of dried weight.

#### 2.1.2. Method Validation

Table 1 shows the HPLC-PDA method validation for oleamide detection. The chromatograms of the standard solution and the WCF test solution were compared to verify the separation of the peak corresponding to the standard compound. The results show that the peak was separated as a single peak without interference from other substances, and the retention times of the standard solution and the test solution peaks were identical (Figure 1). In addition, the ultraviolet (UV) spectra of the standard solution and the WCF test solution were measured. The results demonstrate identical spectra with a similarity index of 999, confirming the specificity of the analytical method. The coefficient of determination (R^2^) of the calibration curve, fit by analyzing three times in the measured concentration range of 5–100 μg/mL, showed a high linearity of 0.999. The detection limit was calculated using the slope and standard deviation (y-intercept) of the calibration curve obtained through linearity verification. The limit of detection (LOD) was 0.37 ± 0.00 μg/mL, and the limit of quantitation (LOQ) was 1.12 ± 0.00 μg/mL. The coefficient of variability (CV%) of the measurement value was analyzed three times with a standard solution prepared at a concentration of 20 μg/mL, and it was calculated to be below 2.5%. It is below the limit as per the recommendations of the Association of Official Analytical Chemists (AOAC) guidelines [18]. To evaluate the recovery of oleamide, it was used in test solutions according to concentration, mixed with WCF, and analyzed three times each. As a result, the recovery of the oleamide standard compounds at concentrations of 5, 10, and 20 μg/mL was 100 ± 5%. These results suggest that the recovery rates are high, falling within the AOAC recovery guidelines of 90–107% [18].

### 2.2. Effect of WCF on Activation of T Cells in Whole Blood of OVA-Induced Mice

**Figure 2:**
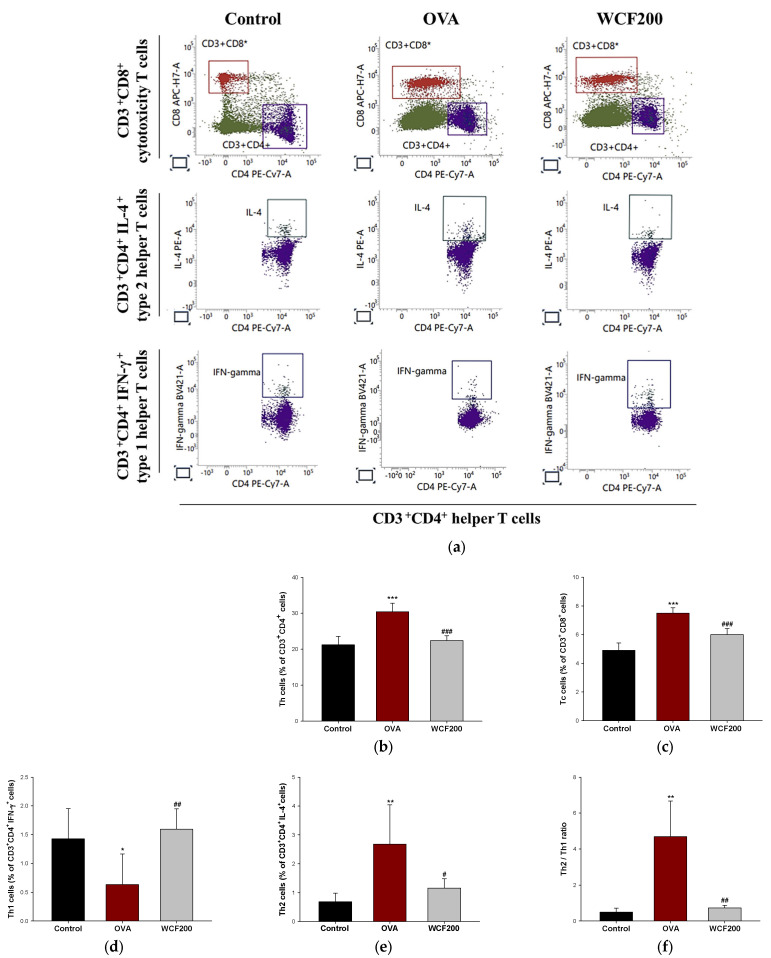
Effect of water extract of *Codium fragile* (WCF) on activation of T cells in whole blood of ovalbumin (OVA)-induced mice. Representative FACS plots (**a**), proportion of CD3^+^CD4^+^ as Th cells (**b**), and CD3^+^CD8^+^ as Tc cells (**c**). Th cell identification was performed by detecting intracellular cytokines IFN-ɣ and IL-4. Proportion of CD3^+^CD4^+^ IFN-γ^+^ as Th1 cells (**d**) and CD3^+^CD4^+^ IL-4^+^ as Th2 cells (**e**), and Th2/Th1 ratio (**f**). Data are presented as mean ± SD (*n* = 5). * *p* < 0.05, ** *p* < 0.01, and *** *p* < 0.001: OVA group vs. control group; # *p* < 0.05, ## *p* < 0.01, and ### *p* < 0.001: WCF groups vs. OVA group.

The numbers of helper T (Th), cytotoxicity T (Tc), and Th1/Th2 cells in the whole blood were detected to evaluate respiratory damage caused by inflammation (Figure 2). Th and Tc cells were detected by analyzing CD4 and CD8 positive cells, respectively. They are considered markers of increased inflammation and tissue damage in asthma models. Both CD3^+^CD4^+^ and CD3^+^CD8^+^ T cells were significantly elevated in the OVA group compared with the control group (*p* < 0.001). However, their numbers were decreased in the WCF200 group compared with the OVA group (*p* < 0.001). Th1 and Th2 cells were assessed by measuring CD3^+^CD4^+^IFN-γ^+^ and CD3^+^CD4^+^IL-4^+^ cells, respectively (Figure 2d,e). As a result, CD3^+^CD4^+^IFN-γ^+^ cells decreased (*p* < 0.05) in the OVA group compared to the control group, while CD3^+^CD4^+^IL-4^+^ cells increased (*p* < 0.01). In contrast, WCF200 treatment increased CD3^+^CD4^+^IFN-γ^+^ cells (*p* < 0.01) and reduced CD3^+^CD4^+^IL-4^+^ cells (*p* < 0.05) compared with the OVA group. Based on this, the Th2/Th1 ratio was analyzed by calculating the ratio of CD3^+^CD4^+^IFN-γ^+^ cells and CD3^+^CD4^+^IL-4^+^ cells (Figure 2f). As a result, it was confirmed that it increased in the OVA group (*p* < 0.01) compared with the control group and decreased in the WCF200 group (*p* < 0.01) compared with the OVA group.

### 2.3. Effect of WCF on Allergic Inflammatory Cytokines in Lung Tissues of OVA-Induced Mice

**Figure 3:**
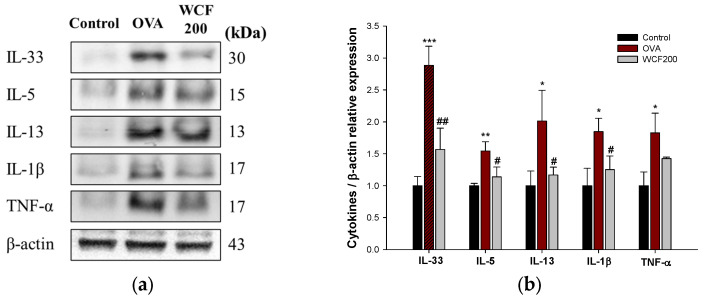
Effect of water extract of *Codium fragile* (WCF) on allergic inflammatory cytokines in lung tissues of ovalbumin (OVA)-induced mice. Western blot images (**a**) and relative expression levels of IL-33, IL-5, IL-13, IL-1β, and TNF-α (**b**). Data are presented as mean ± SD (n = 3). * *p* < 0.05, ** *p* < 0.01, and *** *p* < 0.001: OVA group vs. control group; # *p* < 0.05 and ## *p* < 0.01: WCF groups vs. OVA group.

The expression levels of Th2 and pro-inflammatory cytokines in lung tissues were detected to evaluate the effects of WCF on lung allergic inflammation (Figure 3). Asthma is characterized by a Th2-mediated immune response, which plays a key role in the pathogenesis of allergic airway inflammation by secreting Th2 cytokines such as IL-33, IL-5, and IL-13. IL-1β and tumor necrosis factor (TNF)-α are pro-inflammatory cytokines secreted primarily by macrophages and activate T cells and inflammatory responses. OVA stimulation significantly increased (*p* < 0.05 or less) the expression levels of the Th2 and pro-inflammatory cytokines in lung tissues compared with the control group. However, the expression levels of IL-33, IL-5, IL-13, and IL-1β decreased in the WCF200 group (*p* < 0.05 or less) compared with the OVA group. The WCF200 group did not significantly reduce OVA-induced TNF-α elevation.

### 2.4. Effect of WCF on Number of Leukocytes in Bronchoalveolar Lavage Fluid (BALF) of OVA-Induced Mice

The number of leukocytes in BALF were analyzed to confirm the intensity and nature of lung inflammation (Table 2). In asthma models, leukocytes are an important indicator of the intensity and type of inflammatory response. The leukocytes were classified into eosinophils, lymphocytes, neutrophils, and monocytes. The total leukocyte counts were significantly higher in the OVA group compared to the control group, especially eosinophils, lymphocytes, and neutrophils (*p* < 0.001). In contrast, WCF treatment reduced eosinophils, lymphocytes, and neutrophils as well as total leukocytes (*p* < 0.05 or less). In particular, neutrophils showed a significant difference only in the WCF200 group (*p* < 0.05). There was no significant difference in monocyte counts between all groups.

### 2.5. Effect of WCF on OVA-Specific IgE Levels in BALF and Serum of OVA-Induced Mice

**Figure 4:**
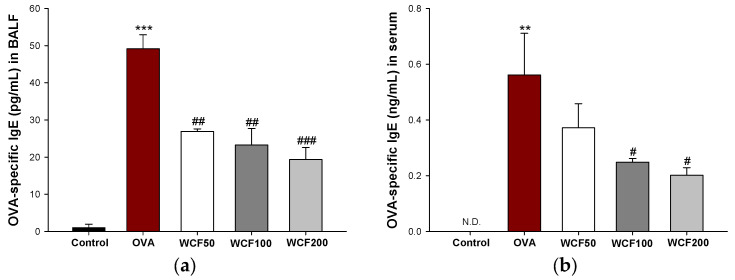
Effect of water extract of *Codium fragile* (WCF) on ovalbumin (OVA)-specific immunoglobulin (Ig)E levels in bronchoalveolar lavage fluid (BALF) (**a**) and serum (**b**) of OVA-induced mice. N.D. means not detected. Data are presented as mean ± SD (n = 5). ** *p* < 0.01 and *** *p* < 0.001: OVA group vs. control group; # *p* < 0.05, ## *p* < 0.01, and ### *p* < 0.001: WCF groups vs. OVA group.

OVA-specific IgE levels in BALF and serum were measured to confirm the induction of an allergic reaction (Figure 4). IgE is a protein that plays an important role in allergic reactions and is a key indicator of OVA sensitization and the challenge model’s success. The OVA-specific IgE level was significantly higher in the OVA group (*p* < 0.001) than in the control group, but WCF groups (*p* < 0.01 or less) down-regulated this level in BALF (Figure 4a). In serum, the OVA-specific IgE level was not detected in the control group, but it was raised in the OVA group (Figure 4b). However, the WCF100 and WCF200 groups were significantly reduced (*p* < 0.05) compared with the OVA group.

### 2.6. Effect of WCF on Histopathological Changes in Lung Tissue of OVA-Induced Mice

**Figure 5:**
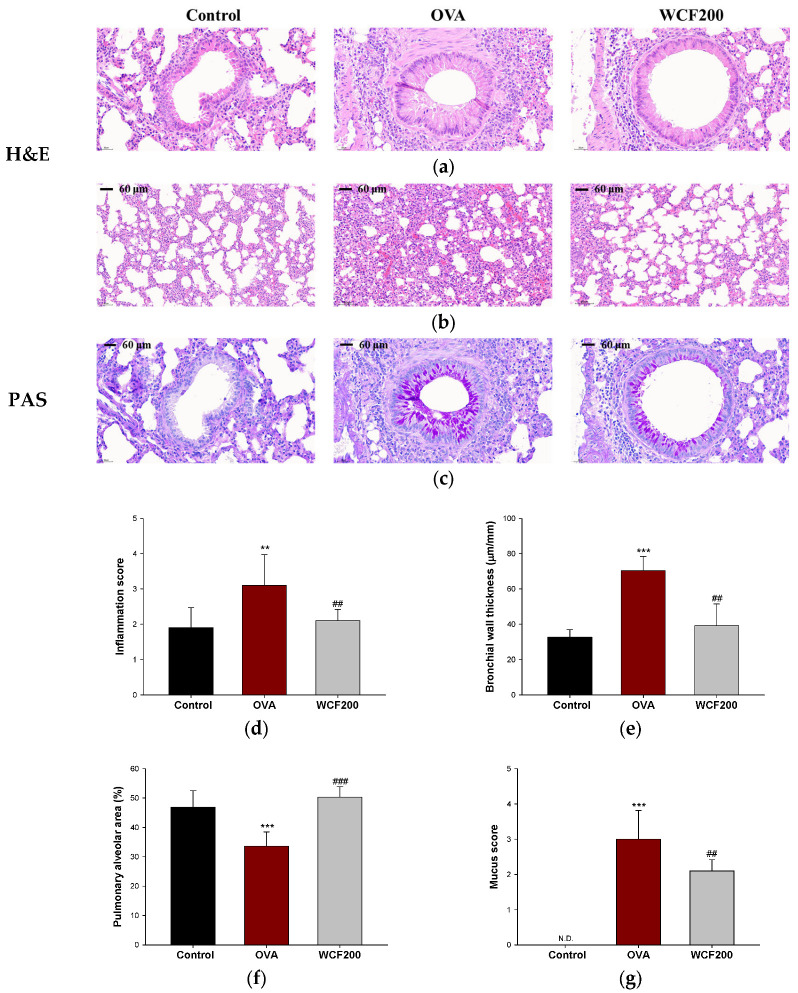
Effect of water extract of *Codium fragile* (WCF) on histopathological changes in lung tissue of ovalbumin (OVA)-induced mice. Representative hematoxylin and eosin (H&E)-stained sections of bronchiole (**a**) and alveoli (**b**), and periodic acid–Schiff (PAS)-stained sections (**c**) of bronchiole in lung tissues. Inflammation score (**d**), bronchial thickness (**e**), pulmonary alveolar area (**f**), and mucus score (**g**). N.D. means not detected. Data are presented as mean ± SD (n = 3). ** *p* < 0.01 and *** *p* < 0.001: control group vs. OVA group; ## *p* < 0.01, and ### *p* < 0.001: OVA group vs. WCF groups.

Histopathological changes were examined to evaluate the effects of WCF on lung inflammation and mucus production in lung tissues. The results of hematoxylin and eosin (H&E) and periodic acid–Schiff (PAS) staining in lung tissues are shown in Figure 5. In asthma models, pathological changes such as inflammatory cell infiltration, increased airway wall thickness, and collagen accumulation are commonly observed. These can affect alveolar size by reducing airway compliance and impeding alveolar airway delivery. Compared with the control group, OVA exposure significantly increased the concentration of inflammatory cells (*p* < 0.01) in the perivascular and peribronchial spaces, as well as the bronchial wall thickness (*p* < 0.001) (Figure 5a,e). Moreover, we confirmed that the alveoli shrunk in the OVA group compared to the control group (Figure 5b,f; *p* < 0.001). Mucus hypersecretion was assessed by detecting PAS-positive cells (Figure 5c,g). As a result, PAS-stained cells were not found in the control group, whereas excessive PAS-positive cells, including goblet cell proliferation, were found in the OVA group (*p* < 0.001). However, WCF intakes restored these histopathological changes such as inflammation scores, bronchial wall thickness, alveolar area, and mucus score compared with the OVA group (*p* < 0.01 or less).

### 2.7. Effect of WCF on Levels of Antioxidant System in Lung Tissues of OVA-Induced Mice

**Figure 6:**
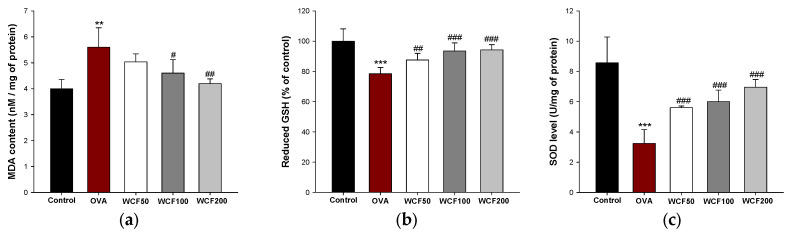
Effect of water extract of *Codium fragile* (WCF) on levels of antioxidant system in lung tissues of ovalbumin (OVA)-induced mice. Malondialdehyde (MDA) content (**a**), reduced glutathione (GSH) (**b**), and *s*uperoxide dismutase (SOD) level (**c**). Data are presented as mean ± SD (n = 5). ** *p* < 0.01 and *** *p* < 0.001: OVA group vs. control group; # *p* < 0.05, ## *p* < 0.01, and ### *p* < 0.001: WCF groups vs. OVA group.

The levels of malondialdehyde (MDA), reduced glutathione (GSH), and superoxide dismutase (SOD) were examined to evaluate the antioxidant effects of WCF in lung tissues (Figure 6). Excessive inflammatory response leads to the formation of reactive oxygen species (ROS) and a decrease in the antioxidant system. To evaluate OVA-induced oxidative stress, the MDA content was determined (Figure 6a). The MDA content was significantly increased in the OVA group compared with the control group (*p* < 0.01). However, the MDA content was significantly decreased in the WCF100 (*p* < 0.05) and WCF200 (*p* < 0.01) groups compared with the OVA group. We evaluated the effect of OVA exposure on the GSH level and SOD activity, which are antioxidants (Figure 6b,c). The GSH level and SOD activity were significantly reduced in the OVA group compared with the control group (*p* < 0.001). Nevertheless, WCF treatment significantly enhanced the levels of antioxidants compared with the OVA group (*p* < 0.01 or less).

### 2.8. Effect of WCF on Activation of Nuclear Factor kB (NF-κB) Pathway in Lung Tissues of OVA-Induced Mice

**Figure 7:**
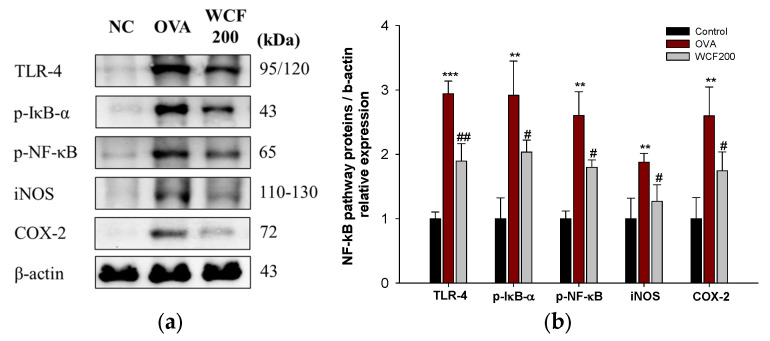
Effect of water extract of *Codium fragile* (WCF) on activation of NF-κB pathway in lung tissues of ovalbumin (OVA)-induced mice. Western blot images (**a**) and relative expression levels of TLR-4, p-IκB-α, p-NF-κB, iNOS, and COX-2 (**b**). Data are presented as mean ± SD (n = 3). ** *p* < 0.01 and *** *p* < 0.001: OVA group vs. control group; # *p* < 0.05 and ## *p* < 0.01: WCF groups vs. OVA group.

The expression levels of Toll-like receptor-4 (TLR-4), phospho-NF-κB inhibitor α (p-IκB-α), p-NF-κB, inducible nitric oxide synthase (iNOS), and cyclooxygenase-2 (COX-2) were detected to investigate the effects of WCF on the NF-κB pathway (Figure 7). The NF-κB pathway is activated by TNF-α and IL-1β, which results in the secretion of many inflammatory mediators from cells. OVA exposure significantly increased the levels of TLR-4, p-IκB-α, p-NF-κB, iNOS, and COX-2 compared with the control group (*p* < 0.01 or less). However, the expression levels of TLR-4, p-IκB-α, p-NF-κB, iNOS, and COX-2 in the WCF200 group were significantly down-regulated compared with the OVA group (*p* < 0.05 or less).

### 2.9. Effect of WCF on Activation of Transforming Growth Factor β (TGF-β)/Smad Pathway in Lung Tissues of OVA-Induced Mice

**Figure 8:**
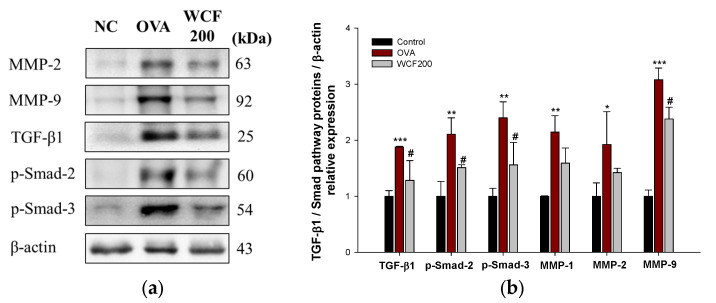
Effect of water extract of *Codium fragile* (WCF) on activation of TGF-β/Smad pathway in lung tissues of ovalbumin (OVA)-induced mice. Western blot images (**a**) and relative expression levels of MMP-2, MMP-9, TGF-β1, p-Smad-2, and p-Smad-3 (**b**). Data are represent mean ± SD (n = 3). * *p* < 0.05, ** *p* < 0.01, and *** *p* < 0.001: OVA group vs. control group; # *p* < 0.05: WCF groups vs. OVA group.

The expression levels of matrix metalloproteinases (MMP)-2, MMP-9, TGF-β1, p-Smad-2, and p-Smad-3 were examined to confirm the effects of WCF on the TGF-β/Smad pathway (Figure 8). Pulmonary fibrosis progression is closely associated with the TGF-β/Smad pathway, which facilitates fibroblast activation and leads to the accumulation of excessive extracellular matrix. OVA exposure significantly up-regulated the levels of TGF-β1, p-Smad-2, p-Smad-3, MMP-1, MMP-2, and MMP-9 compared with the control group (*p* < 0.01 or less). However, the expression levels of TGF-β1, p-Smad-2, p-Smad-3, MMP-2, and MMP-9 in the WCF200 group were significantly down-regulated compared with the OVA group (*p* < 0.05).

### 2.10. Effect of WCF on Activation of Apoptosis Pathway in Lung Tissues of OVA-Induced Mice

**Figure 9:**
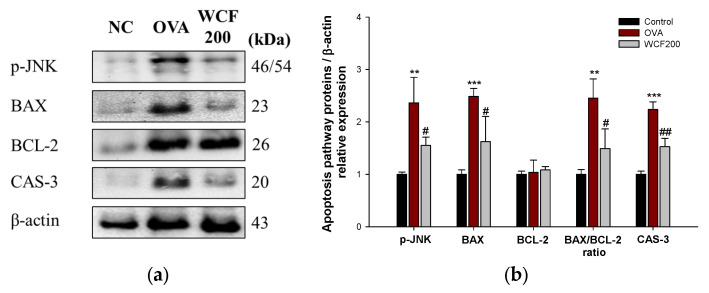
Effect of water extract of *Codium fragile* (WCF) on activation of apoptosis pathway in lung tissues of ovalbumin (OVA)-induced mice. Western blot images (**a**) and relative expression levels of p-JNK, BAX, BCL-2, BAX/BCL-2 ratio, and CAS-3 (**b**). Data are presented as represent mean ± SD (n = 3). ** *p* < 0.01, and *** *p* < 0.001: OVA group vs. control group; # *p* < 0.05 and ## *p* < 0.01: WCF groups vs. OVA group.

The expression levels of phospho-Jun N-terminal kinase (p-JNK), B-cell lymphoma 2 (BCL2), BCL-2 associated X (BAX), and caspase (CAS)-3 were measured to investigate the effects of WCF on the apoptosis pathway (Figure 9). Elevated levels of apoptosis-related proteins indicate OVA-induced lung cell damage. OVA exposure significantly increased the levels of p-JNK, BAX, and CAS-3 compared with the control group (*p* < 0.01 or less). In contrast, the expression levels of p-JNK, BAX, and CAS-3 in the WCF200 group significantly decreased compared with the OVA group (*p* < 0.05 or less). There was no significant difference in BCL-2 expression between all groups. However, the BAX/BCL-2 ratio increased in the OVA group compared with the control group (*p* < 0.01). Conversely, the ratio significantly decreased in the WCF200 group compared with the OVA group (*p* < 0.05).

## 3. Discussion

Allergic asthma is a common respiratory disease that, in response to specific allergens, causes airway inflammation and lung damage [3]. When the airway is exposed to an antigen, the antigen is recognized by antigen-presenting cells and T cells are activated. Specifically, the Th2 immune response is activated, promoting eosinophil recruitment and IgE synthesis by leasing IL-4, IL-5, and IL-13 from bronchial epithelial cells [4]. Subsequently, airway inflammation intensifies, promoting excessive extracellular matrix (ECM) accumulation, leading to airway fibrosis and lung damage [19]. Asthma can be managed through early diagnosis and treatment, but long-term use of current drugs may cause side effects [9]. Accordingly, there is a growing interest in natural products as safer therapeutic alternatives [6]. *C. fragile* has been reported to have immunomodulatory activity as a source of various biologically active compounds, including oleamide [12,16,20]. Therefore, we aimed to evaluate the protective respiratory function effects of WCF in OVA-induced allergic bronchial disorders in mice.

In this study, HPLC-PDA analysis was used to analyze oleamide in WCF. Typically, oleamide, a fatty acid amide derived from the amide form of oleic acid, is analyzed using GC-MS. However, we aimed to develop and validate an HPLC-PDA method for industrial applicability for analyzing oleamide. Consequently, in compliance with AOAC standards, we established an HPLC-PDA method for quantifying oleamide in *C. fragile*. WCF contains significant amounts of oleamide, which has been reported to exhibit anti-allergic, anti-inflammatory, and antioxidant effects [13,15,16,17]. A previous study has shown that oleamide, a bioactive compound found in the leaves of *Anacardium occidentale*, exhibits bronchodilatory and anti-inflammatory effects [21]. Furthermore, it was reported that oleamide reduced the increased mRNA protein expression of inflammatory mediators COX-2 and iNOS in lipopolysaccharide (LPS)-treated RAW 264.7 cells [17]. Therefore, we expected that WCF containing oleamide would show a positive effect on respiratory function in OVA-induced allergic bronchial disorders in mice.

OVA is the most commonly used irritant to trigger eosinophilic asthma. The OVA-induced mouse model was developed with typical asthma features, including moderate airway hyperresponsiveness, excessive collagen deposition, and inflammation [22]. In this study, OVA exposure increased CD3^+^CD4^+^ and CD3^+^CD8^+^ T cells and CD3^+^CD4^+^IL-4^+^ cells in the blood, which was regulated by WCF treatment. The activation of Th2 cells drives the inflammatory response by promoting eosinophil recruitment and IgE production through the release of IL-33, IL-4, IL-5, and IL-13 [22]. Consistent with this mechanism, our results also showed an increase in the number of leukocytes, including eosinophils, as well as OVA-specific IgE levels in the BALF. Th2 cytokines can induce various downstream responses in asthma as well. Specifically, IL-13, in conjunction with IL-4, plays a role in Th2 cell differentiation and contributes to fibrosis by promoting mucin overproduction and thickening of the basement membrane [23]. In this study, severe inflammatory infiltrates around the bronchi, increased bronchial wall thickness, and PAS-positive goblet cells in the lung tissues of OVA-exposed mice were observed. However, WCF intake ameliorated these pathological and histological symptoms of asthma. Therefore, these results suggest that WCF may be used as a preventive agent against OVA-induced respiratory injury by modulating type 2 immune responses.

The molecular mechanisms by which WCF exerts beneficial effects on OVA-induced inflammation and airway remodeling were identified by measuring oxidative stress markers and protein expression in the lung tissues of mice. Recent studies have reported that mast cells regulate allergic airway inflammation through increased TLR4-mediated Th2 cytokine production [24,25]. ROS production increases while antioxidant activity declines under oxidative stress caused by excessive inflammatory responses [26]. ROS accumulation can stimulate NF-κB activation, thereby promoting the production of pro-inflammatory cytokines such as IL-1β and TNF-α [12,25]. Therefore, the TLR-4/NF-κB axis plays an essential role in modulating immune responses and is considered a major contributor to airway inflammation in asthma [27]. In this study, WCF significantly down-regulated the TLR4/NF-κB pathway and oxidative stress stimulated by OVA. Similar findings were observed in an earlier study, where oleamide and *C*. *fragile* were found to reduce the inflammatory response in LPS-treated RAW264.7 cells by down-regulating NF-κB signaling [17,20]. Furthermore, *C. fragile* reduced inflammatory enzymes such as COX-2 and iNOS against ultraviolet B-induced oxidative stress in HaCaT cells [14]. These results suggest that WCF exhibits an improving effect on respiratory function through the suppression of TLR-4/NF-κB pathway-mediated immune response and oxidative stress.

Airway remodeling refers to the pathological process in which the airway structure is altered and remodeled due to persistent inflammation and damage from chronic asthma or chronic lung disease [19]. In the asthma model, this is associated with thickening of the airway walls due to tissue fibrosis and excessive mucus production. Neutrophils secrete MMP-8 and MMP-9, which activate TGF-β and promote the decomposition of lung tissue and fibrosis around the small airways [28]. TGF-β1 activates Smad-2/3 and forms a complex with Smad-4 to increase the expression of fibrosis-related genes such as α-smooth muscle actin, collagen, and fibronectin [29]. A previous study demonstrated that OVA exposure increases the TGF-β1/Smad pathway, highlighting it as a critical regulator of airway remodeling [19]. Another previous study reported that IL-13 may interact with TGF-β1 to induce pulmonary fibrosis [23]. Additionally, they explained that blocking the signaling of IL-4 or IL-13 could prevent fibrosis while maintaining anti-inflammatory function. Similarly, our study found that the TGF-β1/Smad pathway was up-regulated along with the increase in neutrophils and IL-13 in the OVA group, which was consistent with the histological analysis data. However, WCF intake alleviated MMPs and TGF-β1/Smad pathway expressions. Therefore, these results suggest that the anti-inflammatory effect of WCF is related to the suppression of pulmonary fibrosis.

When ECM degradation is excessive, movement of inflammatory cells is facilitated, which amplifies the inflammatory response and damages the alveolar structure. Consequently, there is a functional loss of lung tissue [30]. Moreover, the increased Th2 immune response caused by OVA can increase oxidative stress and activate the endogenous apoptosis pathway [23]. Increased oxidative stress activates JNK, which increases the pro-apoptotic protein BAX and activates caspases, thereby inducing apoptosis [31]. Indeed, it was reported that BAX and CAS-3 increased, and BCL-2 decreased in idiopathic pulmonary fibrosis patients [32]. This study confirmed that OVA treatment increased apoptosis proteins. These results are thought to be due to complex interactions between inflammation and oxidative stress. However, by down-regulating the apoptosis pathway, WCF treatment ameliorated OVA-induced lung cell damage. Therefore, these results demonstrate that WCF has a potent anti-apoptotic effect based on anti-inflammatory and antioxidant effects that may protect against OVA-induced respiratory damage.

## 4. Materials and Methods

### 4.1. Materials and Reagents

High-performance liquid chromatography (HPLC)-grade acetonitrile (ACN), methanol, and water were purchased from Thermo Fisher Scientific (Waltham, MA, USA). Oleamide (08393), OVA (A5503), and aluminum hydroxide (239186) were purchased from Sigma-Aldrich (St. Louis, MO, USA). Antibodies of BV786 anti-mouse CD3e (564379), APC-H7 anti-mouse CD8 (560182), PE anti-mouse IL-4 (562044), and BV421 anti-mouse interferon-gamma (IFN-γ) (563376) and intravenous (IV) catheter (382434; 20 gauge, 1.16 inch) were purchased from BD Biosciences (Franklin Lakes, NJ, USA). PE-Cy7 anti-mouse CD4 (100421) antibody and mouse OVA-specific IgE ELISA kit (439807) were purchased from BioLegend (San Diego, CA, USA).

### 4.2. Preparation of WCF

*C. fragile* collected from the coast of Wando-gun (Republic of Korea) was purchased from a local store in July 2024 and washed with tap water until the salinity content was zero. Then, it was dried in a dry oven at 45 °C for 30 h, and its moisture content was kept under 6–9%. The dried *C. fragile* was extracted twice with water (25-fold volume) by reflux at 100 °C for 7 h. Then, the extracts were filtered, concentrated, and then freeze-dried to obtain WCF.

### 4.3. HPLC with Photodiode Array Detection (HPLC-PDA) Analysis

Oleamide is a bioactive and marker compound of C. fragile. HPLC analysis was conducted to determine oleamide content in WCF and to validate the analytical method. WCF powder was dissolved in 100% methanol to a concentration of 5 mg/mL and extracted by sonication for 30 min. The extract was filtered using a PTFE 0.45 mm syringe filter (Whatman, Maidstone, UK). Oleamide standard sample was also prepared in the same method as concentrations of 5, 10, 20, 50, and 100 μg/mL. The filtrates were analyzed using an HPLC-PDA system (Ultramate 3000 series, Thermo Fisher Scientific) with a YMC-Triart C_18_ column (150 × 4.6 mm, 5 μm particle size, YMC, Seongnam, Republic of Korea) at 40 °C. Mobile phase solvent was prepared with 0.1% formic acid in water (A) and 0.1% formic acid in ACN (B). The solvent condition was set to isocratic elution with 80% B solvent at a flow rate of 1 mL/min for 30 min. The UV spectra were recorded at 203 nm.

### 4.4. Method Validation

Method validation was used to confirm the accuracy of the analytical method described above. To validate the analytical method for oleamide quantification in WCF, specificity, linearity, sensitivity, accuracy, precision, and recovery were assessed according to the methodology outlined in a prior report [33].

### 4.5. Animal Experiment Design

#### 4.5.1. Animals

All animal experiments were performed in accordance with the regulations approved by the Institutional Animal Care and Use Committee of Gyeongsang National University (Approval No. GNU-240405-M0076) on 5 April 2024. Female BALB/c mice (4 weeks old) were purchased from Koatech (Pyeongtaek, Republic of Korea). Mice were raised under a stable temperature (22 ± 2 °C) and humidity (55 ± 5%), and a 12 h light/dark cycle condition was used in a semi-specific pathogen-free laboratory.

#### 4.5.2. Immunization and Treatment

**Figure 10:**
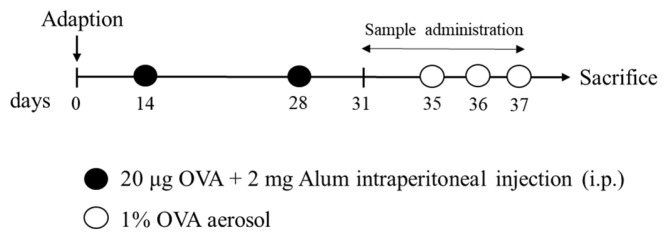
Experimental procedure for ovalbumin (OVA)-induced asthma model and treatment with water extract of *Codium fragile* (WCF).

After a week of adaptation, mice were randomly assigned into five groups (n = 15/group): (1) normal control, (2) OVA-induced asthma model group (OVA), (3) low dose of WCF treatment (WCF50, 50 mg/kg of body weight (B.W.)), (4) middle dose of WCF treatment (WCF100, 100 mg/kg of B.W.), and (5) high dose of WCF treatment (WCF200, 200 mg/kg of B.W.). As previously described [34], the sensitization and challenge schedule are shown in Figure 10. Briefly, mice were immunized with 20 μg OVA emulsified in 2 mg aluminum hydroxide intraperitoneally in a total volume of 0.2 mL on days 14 and 28, while the control group received the same volume of saline. Mice were challenged with the aerosolized 1% (*w*/*v*) OVA (0.25 mL/min) by using a nebulizer (Omron, Kyoto, Japan) for 30 min from days 35 to 37. Mice in the WCF treatment group were treated orally with WCF once daily for one week starting on day 31. The control and OVA groups received drinking water instead. Mice were anesthetized with CO_2_ on day 38, followed by blood collection via the abdominal aorta.

### 4.6. Flow Cytometry Fluorescence-Activated Cell Sorting (FACS) Analysis

Sample preparation for flow cytometry analysis was performed as previously described [31]. Briefly, whole blood was stained with the following antibodies to detect T cell populations and intracellular cytokines: BV786 anti-mouse CD3e, PE-Cy7 anti-mouse CD4, APC-H7 anti-mouse CD8, PE anti-mouse IL-4, and BV421 anti-mouse IFN-γ. The stained cells were analyzed by three-color flow cytometry using a BD FACSLyric device (BD Biosciences), and data were processed using BD FACSuite software (BD Biosciences).

### 4.7. Cell Counting in BALF

BALF was obtained by injecting and aspirating 0.7 mL of sterile phosphate-buffered saline (PBS) through a polyethylene IV catheter inserted into the trachea as described previously with some modifications [35]. This process was repeated twice, and the collected BALF was centrifuged at 300× *g* for 5 min at 4 °C. The cell pellet was used to count leukocytes after resuspending in PBS. Total leukocyte, eosinophils, lymphocytes, neutrophils, and monocytes were analyzed in BALF using an automated cell counter (SYSMEX XN-V, Sysmex Corporation, Kobe, Japan).

### 4.8. OVA-Specific IgE Level

The blood samples were centrifuged at 10,000× *g* for 10 min at 4 °C, and BALF was centrifuged at 300× *g* for 5 min at 4 °C [31]. The resulting supernatant was used for biochemical analysis. OVA-specific IgE levels in BALF and serum were determined using an ELISA kit according to the manufacturer’s recommendations.

### 4.9. Histopathological Analysis

Lung tissues were fixed in 10% neutral buffered formalin. The fixed lung tissues were sectioned into specimens of appropriate size and approximately 2–3 mm in thickness for histological processing. Then, tissue blocks were made and sectioned into slices approximately 3 µm thick using a Finesse ME Microtome (Thermo Fisher Scientific). Sections were then slide-mounted, dried, deparaffinized, rehydrated, and rinsed with distilled water. The sections were stained with H&E and PAS to assess lung inflammation, bronchial wall thickness, and goblet cell hyperplasia. As described in a previous study [36], inflammation and mucus scores were evaluated on a subjective scale from 0 to 4, and data were collected from five regions per tissue. Briefly, inflammatory cell infiltration was scored on a scale from 0 to 4, where 0 = normal; 1 = few cells; 2 = a one-cell-layer ring; 3 = a 2–4-cell-layer ring; and 4 = a ring more than four cells deep. Goblet cell hyperplasia in the bronchi and bronchioles was assessed as follows using a five-point scale based on the percentage of PAS-positive cells: 0 = <0.5%, 1 = <25%, 2 = 25–50%, 3 = 50–75%, and 4 = >75%. The bronchial wall thickness was calculated by dividing the values measured in four different directions of the bronchiole tube (μm) by the basement membrane length (mm). The alveolar size was assessed by measuring alveolar area (%) using the Image J program (NIH, Bethesda, MD, USA). The alveolar area and bronchial wall thickness data were collected from three regions per tissue.

### 4.10. Measurement of SOD, Reduced GSH, and MDA Levels

Antioxidant parameters in lung tissue were measured as previously described [31]. Briefly, lung tissues were homogenized using a bullet blender (New York, NY, USA). The homogenates were centrifuged to collect pellets and supernatants for the determination of SOD, reduced GSH, and MDA levels. SOD activity was determined using a commercially available kit following the protocol provided by the manufacturer (Dojindo Laboratories, Kumamoto, Japan).

### 4.11. Western Blotting

Sample preparation and experimental procedures followed previously described methods [31]. Briefly, lung tissues were homogenized with lysis buffer containing 1% (*v*/*v*) protease inhibitor using an ultrasonic homogenizer on ice. The obtained homogenates were centrifuged at 13,000× *g* for 10 min at 4 °C. The amount of protein in supernatants was quantified using the Bradford reagent (Bio-Rad, Hercules, CA, USA). Protein samples were subjected to 8–12% SDS polyacrylamide gel and transferred to polyvinylidene fluoride (PVDF) membranes (Millipore, Burlington, MD, USA). To prevent nonspecific binding, membranes were incubated in 5% (*v*/*v*) skim milk for 1 h at room temperature, followed by overnight incubation with primary antibodies at 4 °C. The primary antibodies were removed, and membranes were incubated with secondary antibodies for 2 h at room temperature. Protein signals were detected by ECL and captured using the iBright CL1000 imaging system (Thermo Fisher Scientific). Details of primary and secondary antibodies are shown in Table S1.

### 4.12. Statistical Analysis

Data were presented as mean ± standard deviation (SD), and ‘n’ refers to the number of mice in each group. Using the SAS program (Ver. 9.4 SAS Institute, Cary, NC, USA), a one-way ANOVA followed by Tukey’s honest significant difference test were conducted to analyze the results. A two-tailed Student’s *t*-test was used to test the statistical difference between the two groups. Differences were considered statistically significant at *p* < 0.05. The normality of data distribution was confirmed using the Shapiro–Wilk test (https://www.statskingdom.com).

## 5. Conclusions

In conclusion, our study demonstrates that WCF improved respiratory dysfunction by inhibiting pulmonary inflammation, oxidative stress, and fibrosis in OVA-induced asthma mice. Its mechanism may be related to regulating the Th2 immune response and the NF-κB and TGF-β1 pathways by oleamide-containing WCF, which suggests that WCF can improve allergic bronchial disorders. These findings suggest that WCF could serve as a potential preventive for allergic respiratory diseases such as asthma. However, as the present study lacks direct comparison with conventional therapies, further research is needed to validate its therapeutic relevance and to identify the major active compounds responsible for its effects.

## Figures and Tables

**Table 1:** Validation parameters of high-performance liquid chromatography with photodiode array detection (HPLC-PDA) analysis for oleamide.

Parameters	Oleamide (9-octadecenamide)		
Linearity range (μg/mL)	5–100		
Regression equation	y = 0.6112x + 0.4277		
Correlation coefficient (R^2^)	0.9998		
Intra-day precision (%) ^1^	0.14		
Inter-day precision (%) ^1^	0.84		
LOD (μg/mL) ^2^	0.37 ± 0.00		
LOQ (μg/mL) ^2^	1.12 ± 0.00		
Recovery rate (%) ^3^	Concentration (μg/mL)		
	5	10	20
	96.64 ± 1.10	99.62 ± 0.91	100.72 ± 1.10

The results are expressed as the mean ± SD (n = 3). ^1^ Oleamide precision was assessed via three replicates on one day and over three days. Intra- and inter-day variability are expressed as %CV, calculated by (SD/mean) × 100. ^2^ The LOD and LOQ were calculated by using the equations LOD = 3.3 × δ/S and LOQ = 10 × δ/S. ‘δ’ is the standard deviation of the peak areas of the oleamide, and ‘S’ is the slope of the corresponding calibration curve. ^3^ Accuracy was assessed through a recovery evaluation performed via the standard addition approach.

**Table 2:** The number of leukocytes in the bronchoalveolar lavage fluid (BALF) of ovalbumin (OVA)-induced mice.

Groups	Total Leukocytes	Leukocyte Classification Unit: 10^5^/mL			
		Eosinophils	Lymphocytes	Neutrophils	Monocytes
Control	2.70 ± 0.41	N.D.	N.D.	N.D.	2.67 ± 0.38
OVA	7.64 ± 0.43 ***	3.31 ± 0.88 ***	1.15 ± 0.19 ***	0.86 ± 0.31 ***	2.30 ± 0.43
WCF50	5.26 ± 0.56 ^##^	1.62 ± 0.23 ^##^	0.68 ± 0.19 ^##^	0.68 ± 0.22	2.29 ± 0.25
WCF100	5.16 ± 1.94 ^#^	1.96 ± 0.84 ^#^	0.64 ± 0.20 ^##^	0.56 ± 0.22	2.13 ± 1.00
WCF200	5.30 ± 0.82 ^#^	1.76 ± 0.25 ^##^	0.63 ± 0.12 ^###^	0.42 ± 0.09 ^#^	2.50 ± 0.46

N.D. means not detected. The results are expressed as the mean ± SD (n = 5). *** *p* < 0.001: OVA group vs. control group; ^#^ *p* < 0.05, ^##^ *p* < 0.01, and ^###^ *p* < 0.001: WCF groups vs. OVA group.

## Data Availability

The data presented in this study are available upon request from the corresponding author.

## Supplementary Materials

The following supporting information can be downloaded at https://www.mdpi.com/article/10.3390/md23050221/s1, Table S1. Information on primary and secondary antibodies used in Western blotting.
