# Supplementary material for: Codium fragile Extract Ameliorates Respiratory Function by Controlling Allergic Inflammation in Ovalbumin-Induced Bronchial Disorders in Mice
Source: Mar Drugs. 2025 May 21;23(5):221. doi: 10.3390/md23050221 (PMC12113249; doi:10.3390/md23050221)
Supplement: Supplementary file 1 [file marinedrugs-23-00221-s001.zip › marinedrugs-3621200-supplementary.pdf]

Table S1. Information on primary and secondary antibodies used in western blot.

Gene name	Cat No.	Company
β-actin	sc-69879	Santa Cruz Biotech (Dallas, TX, USA)
IL-33	sc-517600	Santa Cruz Biotech
IL-5	sc-398334	Santa Cruz Biotech
IL-13	sc-393365	Santa Cruz Biotech
IL-1β	sc-4592	Santa Cruz Biotech
TNF-α	sc-33639	Santa Cruz Biotech
TLR-4	sc-52962	Santa Cruz Biotech
p-IkB-α	sc-8404	Santa Cruz Biotech
p-NF-κB	sc-136538	Santa Cruz Biotech
iNOS	sc-7271	Santa Cruz Biotech
COX-2	sc-37681	Santa Cruz Biotech
MMP-2	sc-13595	Santa Cruz Biotech
MMP-9	sc-13520	Santa Cruz Biotech
TGF-β1	sc-130348	Santa Cruz Biotech
p-Smad-2	#3108	Cell Signaling Tech (Danvers, MA, USA)
p-Smad-3	sc-517575	Santa Cruz Biotech
p-JNK	sc-6254	Santa Cruz Biotech
BAX	sc-7480	Santa Cruz Biotech
BCL-2	sc-7382	Santa Cruz Biotech
CAS-3	sc-56053	Santa Cruz Biotech
Goat-anti-mouse IgG	AP124P	Sigma-Aldrich (St. Louis, MO, USA)
Goat-anti-rabbit IgG	#7074	Cell Signaling Tech
